# Effect of volatile compounds produced by *Ralstonia solanacearum* on plant growth promoting and systemic resistance inducing potential of *Bacillus* volatiles

**DOI:** 10.1186/s12870-017-1083-6

**Published:** 2017-08-02

**Authors:** Hafiz Abdul Samad Tahir, Qin Gu, Huijun Wu, Waseem Raza, Asma Safdar, Ziyang Huang, Faheem Uddin Rajer, Xuewen Gao

**Affiliations:** 1 Department of Plant Pathology, College of Plant Protection, Nanjing Agricultural University, Key Laboratory of Integrated Management of Crop Diseases and Pests, Ministry of Education, Weigang No.1, Nanjing, 210095 People’s Republic of China; 2 Plant Pathology section, Tobacco Research station, Pakistan Tobacco Board, Ministry of Commerce, Govt. of Pakistan, Hayatabad, Peshawar, Pakistan

**Keywords:** *Bacillus subtilis*, Growth promotion, Induced resistance, PPO and PAL, VOCs

## Abstract

**Background:**

Microbial volatiles play an expedient role in the agricultural ecological system by enhancing plant growth and inducing systemic resistance against plant pathogens, without causing hazardous effects on the environment. To explore the effects of VOCs of *Ralstonia solanacearum* TBBS1 (*Rs*) on tobacco plant growth and on plant growth promoting efficiency of VOCs produced by *Bacillus subtilis* SYST2, experiments were conducted both in vitro and *in planta.*

**Results:**

The VOCs produced by SYST2 significantly enhanced the plant growth and induced the systemic resistance (ISR) against wilt pathogen *Rs* in all experiments. The SYST2-VOCs significantly increased PPO and PAL activity and over-expressed the genes relating to expansin, wilt resistance, and plant defense while repressed the genes relating to ethylene production. More interestingly, VOCs produced by pathogen, *Rs* had no significant effect on plant growth; however, *Rs*-VOCs decreased the growth promoting potential of SYST2-VOCs when plants were exposed to VOCs produced by both SYST2 and *Rs*. The co-culture of SYST2 and *Rs* revealed that they inhibited the growth of each other; however, the inhibition of *Rs* by SYST2-VOCs appeared to be greater than that of SYST2 by *Rs*-VOCs.

**Conclusion:**

Our findings provide new insights regarding the interaction among SYST2-VOCs, *Rs*-VOCs and plant, resulting in growth promotion and induced systemic resistance against the bacterial wilt pathogen *Rs*. This is the first report of the effect of VOCs produced by pathogenic microorganism on plant growth and on plant growth-promoting and systemic resistance-inducing potential of PGPR strain SYST2.

**Electronic supplementary material:**

The online version of this article (doi:10.1186/s12870-017-1083-6) contains supplementary material, which is available to authorized users.

## Background

Bacterial wilt is a devastating and destructive soil born disease covering a range of nearly 450 crop species, particularly the crops of *Solanaceae* family [1, 2]. Bacterial wilt widely occurs in tropical and subtropical regions of the world [3]. The biological control method is environment-friendly, cost-effective and easily applicable method for the management of soil born plant pathogens. Plant growth promoting bacteria are not only involved in plant growth promotion but are also considered to be the best biocontrol agents, having the potential to suppress the population of pathogenic microorganisms and to induce the systemic resistance in plants against diseases [4]. The mechanisms involved in plant growth promotion comprising nitrogen fixation, secretion of phytohormones, solubilization of minerals [5–7], and production of antimicrobial compounds [8], including volatile organic compounds (VOCs) [9–12]. The VOCs produced by plant growth-promoting rhizobacteria (PGPR) are low molecular weight, gaseous, metabolic compounds emitted from bacterial cells under normal conditions [13], which are active even at low concentrations [14]. The VOCs produced by PGPRs play an expedient role in three ways; by controlling plant pathogens, stimulating plant growth and inducing systemic resistance [12, 15–18]. The mode of action of VOCs has an extra degree of advantage over other biocontrol and growth-regulating mechanisms that VOCs don’t need any physical contact with pathogen or plant parts while most of the other processes involved in controlling phytopathogens and promoting plant growth, require physical contact and close vicinity [4, 5]. Lemfack (2014) reported 300 bacteria and fungi as VOC producers, while 846 VOCs with 5431 synonyms were recorded in the database of volatiles emitted by microorganisms (DOVE-MO) [19].

Numerous bacterial species have been reported to have an influence on plant growth promotion and induction in systemic resistance including *Bacillus, Pseudomonas, Stenotrophomonas, Serratia* and *Arthrobacter* [15, 16, 20, 21]*. Bacillus subtilis* GB03 and *B. amyloliquefaciens* IN937a were reported first time by Ryu (2003) as the producer of plant growth promoting VOCs; 2,3-butanediol and acetoin [15]. A VOC 2-pentylfuran emitted by *Bacillus megaterium* XTBG34 stimulated *Arabidopsis thaliana* growth [16] while 13-tetradecadien-1-ol, 2-butanone and 2-methyl-n-1-tridecene, produced by *Pseudomonas fluorescens* SS101 promoted the growth of *Nicotiana tabacum* [18]. Besides their growth-promoting activity, the VOCs elicit plant tolerance against both biotic and abiotic elements by inducing systemic resistance. The *bacillus* VOCs, 2,3-butanediol have been testified to significantly induce resistance in the *Arabidopsis* plant against the pathogen *Erwinia carotovora sub sp. carotovora* [22] while 3-pentanol and 2-butanone, against the pathogen *Pseudomonas* s*yringae* pv. *lachrymans* [23]*,* a causal agent of bacterial angular leaf spot of cucumber. Similarly, another study reported that *Paenibacillus polymyxa E681* produced tridecane, which significantly induced systemic resistance in plants [17].

A few studies have been reported concerning the mechanism of plant growth promotion and systemic resistance induction triggered by bacterial VOCs. Some researchers publicized that bacterial VOCs can interact with plant hormones by involving in morphogenetic processes, consequently trigger the plant growth promotion [11, 15, 24, 25]. Xie et al. (2009) reported an enhancement in photosynthetic activity and chlorophyll contents, when *Arabidopsis* seedlings were exposed to VOCs of *Bacillus subtilis* GBO3 [26]. Transcriptional analysis of *Arabidopsis* after exposure to *Bacillus subtilis* GBO3-VOCs revealed that VOCs regulated the auxin which resulted in the initiation of growth promotion [24]. Furthermore, VOCs differentially expressed the transcriptional expression of genes relating to ethylene response and ethylene biosynthesis [27].

Among all PGPR species, *Bacillus* species are deliberated as the most efficacious species since they have the capability to produce spores that can persist in adversative environmental conditions [28]. In our previous studies, we identified two VOCs, albuterol and 1,3-propanediole produced by *Bacillus subtilis* SYST2 that promoted the plant growth of tomato [11]. The main objective of this study was to explore the impact of VOCs produced by SYST2, in-vitro and *in-planta*, on plant growth promotion and activation of induced systemic resistance in tobacco; in addition, the effect of VOCs produced by pathogen *Ralstonia solanacearum* on the activity of VOCs of PGPR strain SYST2 was also explored.

## Results

### SYST2-VOCs enhance tobacco plant growth while *Rs*-VOCs decrease the efficiency of PGPR strain SYST2

#### In vitro assay

**Fig. 1:**
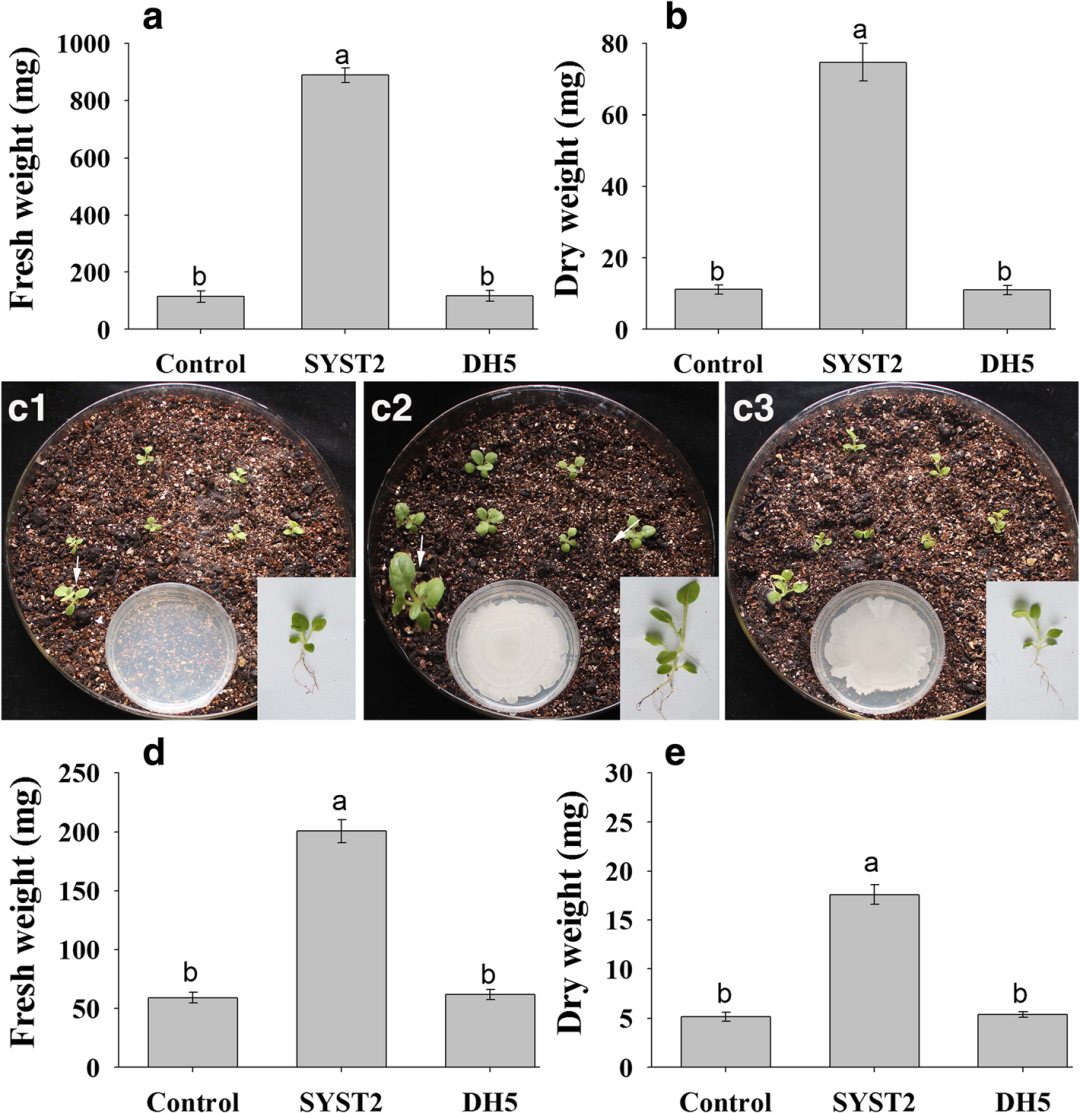
Effect of SYST2-VOCs on Plant growth promotion of tobacco in vitro. For divided plate system (**a** and **b**), seven germinated seedlings were transplanted in one partition and bacteria were spot inoculated on the other partition. For “two plate system”, germinated seedlings were grown in larger plates having soil mixed with vermiculite as a medium for growth, while bacterial strains were inoculated in small plates. The effect of SYST2-VOCs on plant growth was observed by recording fresh green weight **(a)** and dry weight **(b)** after two weeks of exposure. In “two plate system” effect was observed visually which showed different growth in control (**C1**), exposed to SYST2-VOCs (**C2**), and exposed to DH5-VOCs (**C3**). Fresh weight of tobacco seedlings (**d**) and dry weight (**e**) of seedlings were recorded after 10 days in “two plate system”. Error bars indicate the standard deviation of the mean (*n* = 5). Different lower case letters above the columns represent significant differences between treatments at *P* = 0.05. Experiments were repeated three times with similar results

To determine the effect of SYST2-VOCs on plant growth in vitro, tobacco seedlings were grown in divided Petri plates using MS medium for plant growth, and in “two plate system” using soil as a medium for plant growth. In divided plate system, data were taken after 14 days and a significant increase was observed in fresh green weight and dry weight of tobacco plants exposed to SYST2-VOCs as compared to the water control and DH5α control. The VOCs emitted by SYST2 enhanced both the fresh and dry weights of tobacco plants significantly, by 7.7-fold, compared to the controls (Fig. 1a and b). Similarly, in two plate system, the SYST2-VOCs significantly enhanced both fresh and dry weights of tobacco plants by 3.5 fold, compared to controls (Fig. 1C1-1E). No significant differences were noted when the results of plants exposed to the VOCs of *E. coli* DH5α (negative control) were compared to the water control in both experimental systems (Fig. 1).

**Fig. 2:**
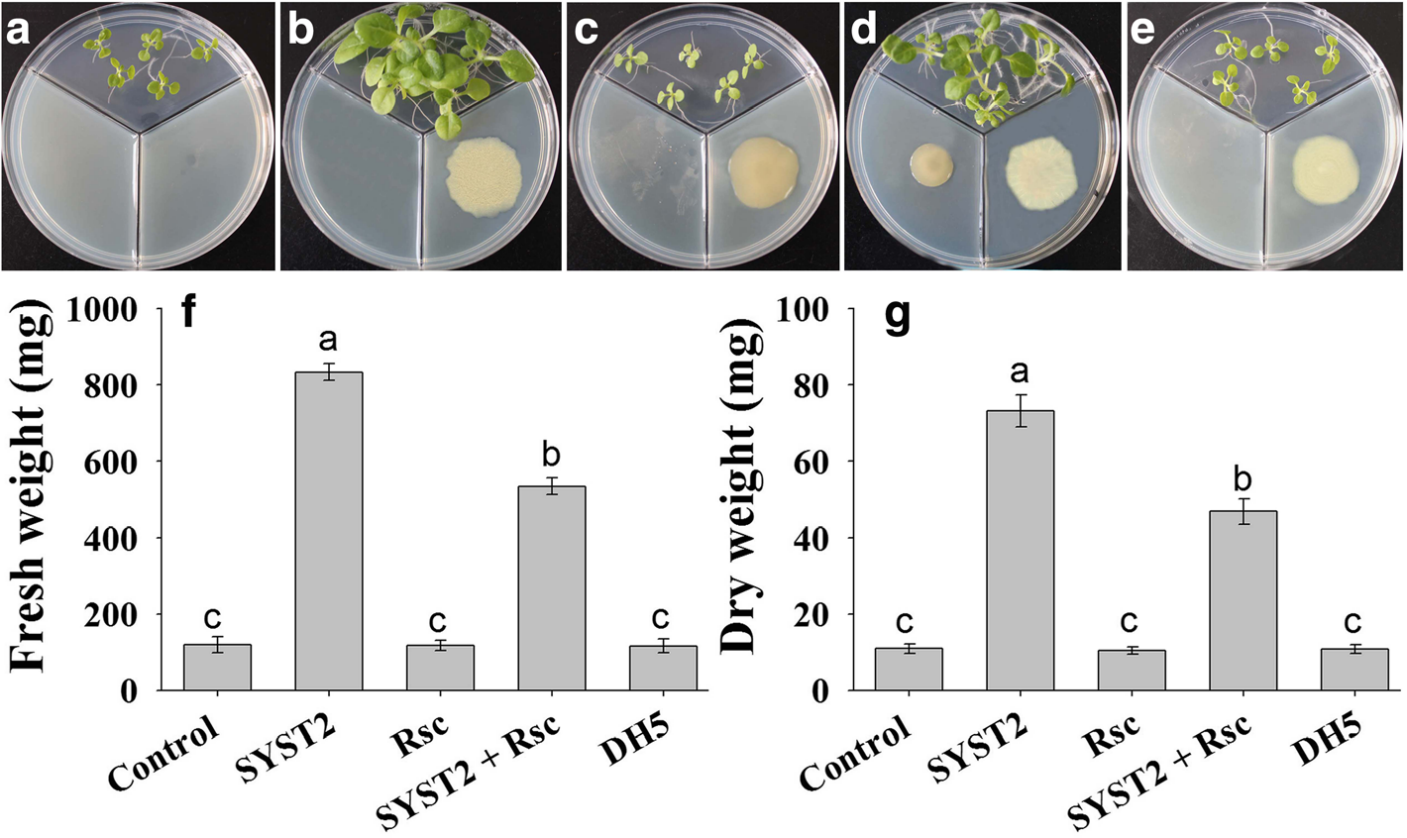
Effect of *Rs*-VOCs on plant growth promoting potential of SYST2-VOCs in vitro. Three partitioned plate system was used to evaluate the effect of *Rs*-VOCs on growth-promoting potential of SYST2-VOCs. After two weeks of exposure to VOCs, observations were recorded; control (**a**), SYST2 only (**b**), *Rs* only, (**c**) SYST2 and *Rs* together (**d**) and DH5α (**e**) as a negative control. Fresh (**f**) and dry weights (**g**) of tobacco seedlings were recorded. Error bars indicate the standard deviation of the mean (*n* = 5). Different lower case letters above the columns represent significant differences between treatments at *P* = 0.05. Experiments were repeated three times with similar results

To examine whether *Rs*-VOCs also affected plant growth or affected the efficiency of PGPR strain SYST2, we created a “three partitioned plate system” using MS medium for plant growth. In one partition tobacco seedlings were grown while in other two partitions, PGPR strain SYST2 or pathogenic strain *Rs* was inoculated. Our results revealed that *Rs*-VOCs had no effect on plant growth and showed similar results compared to control. However, the plates in which both SYST2 and *Rs* were inoculated in two separate partitions, the growth promoting effect of SYST2-VOCs was significantly lower, compared to the treatment where tobacco seedlings were exposed to SYST2-VOCs only. *Rs*-VOCs significantly decreased the growth-promoting potential of SYST2. Almost a 7-fold increase was observed when tobacco seedlings were exposed to SYST2-VOCs only while a 4.5-fold increase was observed when exposed to SYST2-VOCs accompanied with *Rs*-VOCs (Fig. 2).

#### *In planta* assay

**Fig. 3:**
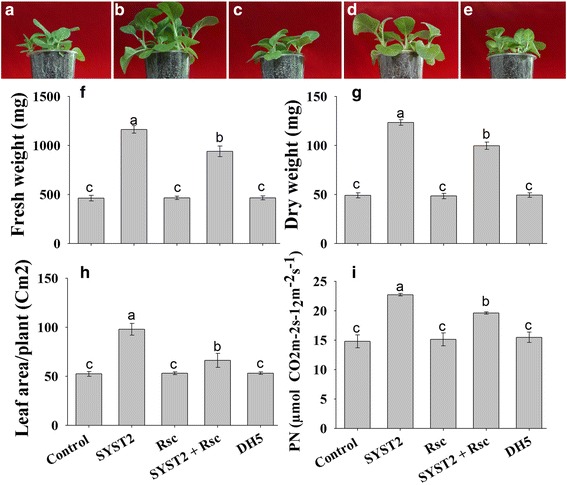
Effect of *Rs*-VOCs on plant growth promoting potential of SYST2-VOCs *in planta.* Five germinated seedlings were grown in pots attached to the inside of jars containing a culture of SYST2, *Rs* or both together. Pots were placed in a growth chamber at 28/22 °C day/night under 16/8-h *light/dark* photoperiod. A clear difference was observed in control (**a**) and after 28 days of exposure to SYST2-VOCs alone (**b**), *Rs*-VOCs (**c**), SYST2 and *Rs*-VOCs together (**d**), and DH5α (**e**). Fresh *green* weight (**f**), dry weight (**g**), leaf area (**h**), and photosynthesis rate (**i**) was recorded after 28 days. Error bars indicate the standard deviation of the mean (*n* = 5). Different lower case letters above the columns represent significant differences between treatments at *P* = 0.05. Experiments were repeated three times with similar results

To investigate the plant growth-promoting potential of VOCs *in planta,* pot experiments was conducted as described by Park et al. [18]. Five tobacco seedlings were transplanted into pots attached to the inside of jars containing a culture of SYST2 or DH5α (for control) at the bottom. After 28 days exposure, the analysis of the data showed a significant increase in the growth of tobacco seedlings in terms of fresh green and dry weight, and leaf area as compared to the water and DH5α controls (Fig. 3a-3h). A significant increase of almost 1.6-fold in photosynthesis rate was also observed after exposure to SYST2-VOCs. Based on our data, we can state that the growth and development of tobacco seedlings was stimulated by *B. subtilis* SYST2 VOCs in all experimental systems. In this experiment, in addition to the evaluation of growth promoting activity of VOCs by SYST2, we evaluated the effect of *Rs*-VOCs on plant growth regulation and on the efficiency of PGPR strain SYST2 *in planta.* For this purpose, in one set we inoculated only *Rs* or SYST2 while in other we used two plates; one inoculated with SYST2 and one inoculated with *Rs. Rs*-VOCs exhibited no effect on plant growth regulation and showed similar results, compared to control. Tobacco seedlings exposed to a combination of both SYST2 and *Rs* had a lesser effect on plant growth promotion, compared to only SYST2-VOCs in terms of all studied parameters; fresh and dry weight, leaf area and photosynthesis rate. A 2.5 fold increase was observed in fresh and dry weight, 1.84 in leaf area and 1.53 in photosynthesis rate when exposed to SYST2-VOCs only while the values were decreased to 2-fold for fresh and dry weight and 1.25-fold both for leaf area and photosynthesis when exposed to SYST2-VOCs accompanied with *Rs*-VOCs (Fig. 3).

### Plant growth promoting activity by specific VOCs produced by SYST2

**Fig. 4:**
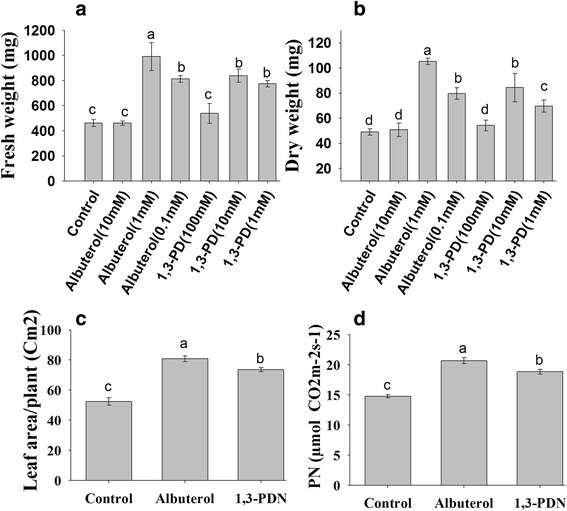
Evaluation of individual VOCs produced by *B. subtilis* SYST2 for plant growth promotion. Tobacco seedlings were exposed to the specific individual VOCs albuterol and 1,3-propanediol dissolved in DMSO at four different concentrations in pots as described above for 28 days at 28/22 °C day/night under 16/8-h light/dark photoperiod. Plant fresh weights **(a)**, dry weights **(b),** leaf area **(c)** and photosynthesis rate **(d)** were determined. Error bars indicate the standard deviation from the mean. Letters above the columns indicate a significant difference at *P* < 0.05. Experiments were replicated three times

All of the compounds produced by SYST2 were tested individually to evaluate their plant growth promoting potential. The two compounds, albuterol and 1,3-propanediol, positively stimulated plant growth promotion as compared to the control. Five germinated tobacco seedlings of equal size were transferred to the plastic pots, fixed on the glass jars while individual compounds were dissolved in DMSO and applied to the plates, which were placed at the bottom of the jar, and incubated at 28/22 °C day/night under 16/8-h light/dark photoperiod for 14 days. The results revealed that albuterol (10 mM & 1 mM) while 1,3-propanediol (100 mM &10 mM) promoted the growth significantly, compared to control. However, no difference was observed when seedlings were exposed to 0.1 mM albuterol and 1 mM 1,3-propanediole, compared to control. A significant difference was observed in fresh green weight, dry weight, leaf area and photosynthesis rate in tobacco plants after exposure to these volatile compounds. Albuterol (1 mM) increased fresh green and dry weight by 2.14-fold, while 1,3-propanediol (10 mM) enhanced fresh green weight by 1.76-fold and dry weight by 1.61-fold as compared to the control (Fig. 4).

### Effect of VOCs produced by *Rs* on the growth of PGPR strain SYST2

**Fig. 5:**
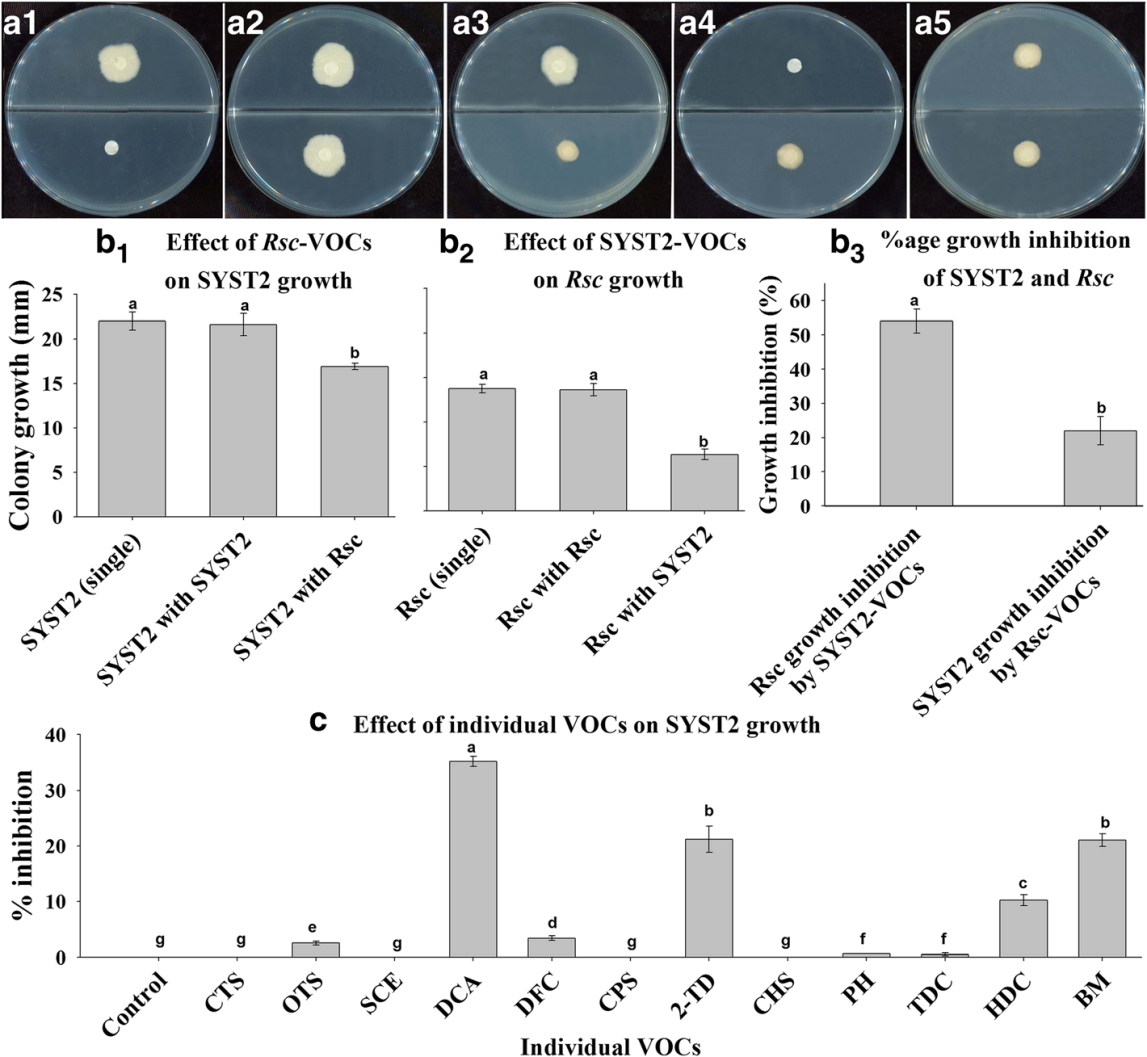
Effect of VOCs produced by *Rs* on the growth of PGPR strain SYST2. Ten μl of (18–24 h) *Rs* culture (1 × 10^7^ CFU ml^−1^) grown in CPG broth was dropped in one partition of I-plate while 10 μl of the liquid overnight-grown culture of SYST2 (1 × 10^8^ CFU ml^−1^) was dropped in the other partition containing modified minimal salt medium (MS). The plates were double sealed and incubated at 28 °C for three days. Only pathogen and only SYST2 were inoculated on control plates. The diameter of *Rs* and SYST2 were measured in mm. Only SYST2 **(a1),** SYST2 with SYST2 **(a2)**, SYST2 with *Rs* **(a3)**, only *Rs* **(a4)** and *Rs* with *Rs* **(a5)**. Effect of VOCs produced by pathogenic strain *Rs* on SYST2 growth **(b1)** and SYST2-VOCs on *Rs* growth was observed **(b2)** and percentage growth inhibition **(b3)** was recorded. Similarly, the effect of specific VOCs produced by *Rs* was evaluated **(c).** Error bars indicate the standard deviation from the mean. Each experiment was performed with five replicates, and the experiment was repeated three times

The VOCs produced by *Rs* reduced the growth-promoting potential of VOCs derived by PGPR strain SYST2 as described in the above experiment. To investigate interaction among VOCs produced by PGPR strain SYST2 and pathogenic strain *Rs,* we examined the effect of *Rs*-VOCs on the growth of SYST2 and vice versa. Results showed that SYST2-VOCs inhibited the colony growth of *Rs* while *Rs*-VOCs inhibited the growth of SYST2; however, this inhibition level was different for SYST2 and *Rs* against each other. SYST2-VOCs inhibited the colony growth of *Rs* more compared to the inhibition of SYST2 growth by *Rs*-VOCs. SYST2-VOCs inhibited the growth of *Rs* up to 54%, but *Rs*-VOCs inhibited the growth of SYST2 up to 22%. VOCs produced by SYST2 or *Rs* have no effect on their own colony growth when cultured same bacteria on both sides of the I-plate (Fig. 5a and b).

To determine which VOCs produced by *Rs*, we collected VOCs, by a combination of HS-SPME and GC-MS. Twelve compounds were identified from *Rs* (see Additional file 1, Table S2), which had relatively high peak areas, e.g., ≥1%, and were not similar to the control. Dichloroacetic acid, 2-ethylhexyl ester (DCA), 2-tetradecanone (2-TD) and butanamide had antibacterial activity against SYST2 and inhibited the growth of SYST2 by 35.23%, 21.23%, and 21.02%, respectively (Fig. 5c).

### SYST2*-*VOCs reduced the wilt disease index by inducing systemic resistance

**Fig. 6:**
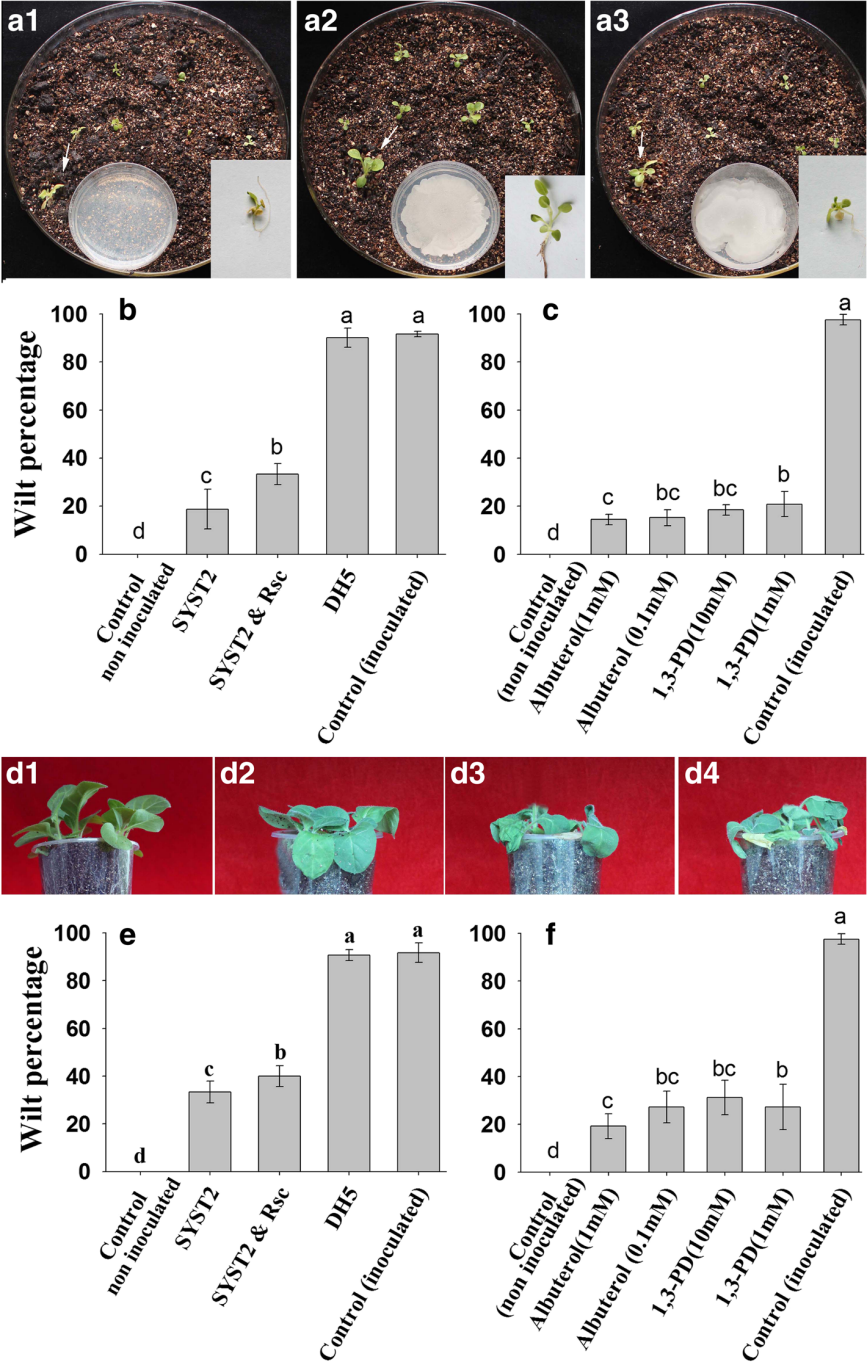
SYST2*-*VOCs reduced the wilt disease index by inducing systemic resistance. For in vitro assay, germinated seedlings were dipped in *Rs* culture and grown in larger plates having soil mixed with vermiculite as a medium for growth. Sterilized water was used as control **(a1)** while PGPR strain SYST2 **(a2)** and DH5α **(a3)** were inoculated in small plates. Wilt index was recorded after 14 days of inoculation after exposure to SYST2-VOCs **(b)** and synthetic VOCs **(c)**. For *in planta* assay, the pots were inoculated with a suspension of *Rs* (at an OD of 0.1 at 600 nm) by dipping the roots in the suspension, except the non-inoculated control **(d1),** and then the seedlings were re-planted in the pots. Small agar plates at the bottom of jars were inoculated with SYST2 **(d2),** DH5α **(d3),** while only sterilized water was used as control **(d4).** Data was recorded after 21 days of inoculation after exposure to SYST2-VOCs **(e)** and synthetic VOCs **(f)**

SYST2-VOCs reduced the wilt disease development significantly (18.66%) when tobacco seedlings were exposed to its VOCs, compared to the control (91.66%), in two plate system. Similarly, individual VOCs albuterol and 1,3-propanediol also reduced the disease development. A disease index of 14.4% and 15.2% was observed by albuterol (1 mM and 0.1 mM) while 18.4% and 20.8% by 1,3-propanediol (10 mM and 1 mM) compared to (97.6%) water control. Reduction in disease development, both by SYST2-VOCs collectively, and by individual VOCs in vitro*,* revealed the mechanism of induced systemic resistance in tobacco against *Rs* by SYST2-VOCs as well as by individual synthetic chemicals (Fig. 6a-c). To demonstrate the effect of VOCs produced by SYST2 on induced systemic resistance at a broader level (in-vivo), we created the *in planta* system as described above. The results revealed that VOCs produced by SYST2 caused a significant reduction in the wilt index, confirming the induced systemic resistance in plants against *Rs*. In the pot experiment, SYST2 significantly reduced the disease and showed only a 33% wilt index, as compared with a 90.66% wilt index in the non-exposed control while albuterol (1 mM) showed only 19% wilt index followed by 1,3-propanediol (1 mM) and albuterol (0.1 mM) with 27% wilt index (Fig. 6 d-f). No significant difference was observed in water control and when plants exposed to DH5α (−ve control).

### Evaluation of the activity of resistance-related enzymes.

**Fig. 7:**
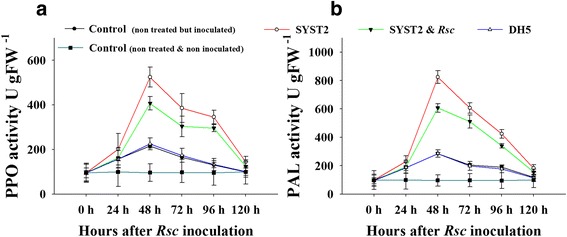
Polyphenol Oxidase (PPO) and Phenylalanine Ammonia Lyases (PAL) Activity. Leaf samples were collected 0 (pre-inoculation), 24, 48, 72, 48, 72, 96 and 120 h after inoculation of *Rs* and activity of PPO **(a)** and PAL **(b)** was measured. Error bars indicate the standard deviation from the mean. Each experiment was performed with five replicates, and the experiment was repeated three times

The effect of SYST2-VOCs alone and in combination with *Rs*-VOCs on the activity of enzymes, relating to resistance (PPO and PAL), was determined in response to inoculation of bacterial wilt pathogen *Rs* in plants that were exposed to VOCs. The activities of PPO and PAL were low and have no significant difference among VOCs-exposed and control plants before pathogen inoculation. A clear increase was observed both in PPO and PAL after pathogen inoculation in plants that were exposed to VOCs when compared to both inoculated and non-inoculated control. The increase in enzyme activity was reached to its highest point at 48 h of inoculation and started to decline after 72 h, reaching its previous position after 120 h of *Rs* inoculation. However, the activities of both the PPO and PAL enzymes were higher in plants exposed to SYST2-VOCs as compared to SYST2 + *Rs*-VOCs. No increase in enzyme activity was observed in non-inoculated control plants while no significant difference was observed between plants exposed to DH5α-VOCs compared to control (Fig 7).

### VOCs affect the transcription of genes related to plant growth regulation and resistance

**Fig. 8:**
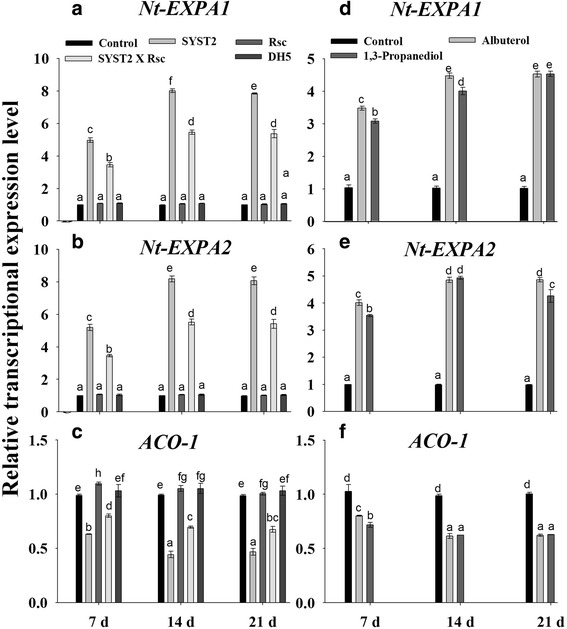
Transcriptional expression profiles of genes involved in growth regulation after exposure to VOCs. qRT-PCR was performed with SYBR Green/Fluorescent qPCR master mix (Takara) on a Roche-480 system (Roche), and *EF-1α* was used as an internal reference. The relative expression levels of *Nt-EXPA1* **(a)** *, Nt-EXPA2* **(b)** and *ACO-1* **(c)** were determined after exposure to VOCs by SYST2 alone or in combination with *Rs*. Similarly, the relative expression levels of *Nt-EXPA1* **(d)** *, Nt-EXPA2* **(e)** and *ACO-1* **(f)** were determined after exposure to synthetic VOCs. Error bars indicate standard errors of the means. Different lower case letters above the error bars represent significant differences according to Duncan’s multiple-range test (*P* ≤ 0.05) using SPSS software (SPSS, Chicago, IL)

**Fig. 9:**
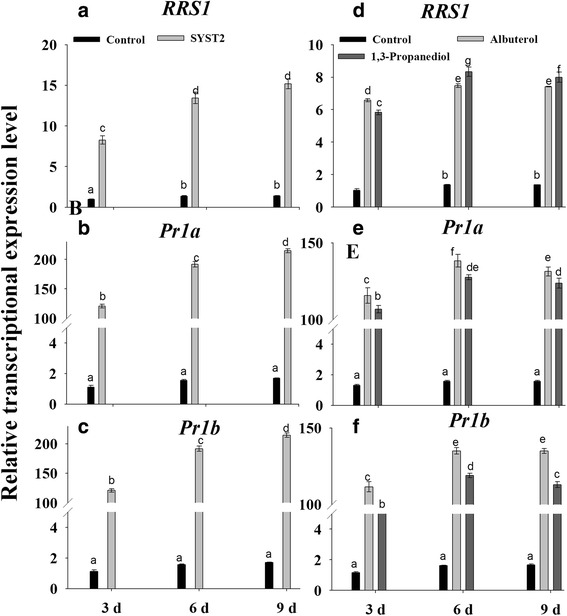
Transcriptional expression profiles of genes involved in resistance after exposure to VOCs. qRT-PCR was performed with SYBR Green/Fluorescent qPCR master mix (Takara) on a Roche-480 system (Roche), taking *EF-1α* was as an internal reference. The relative expression levels of *RRS1* **(a)** *, Pr1a* **(b)** and *Pr1b* **(c)** were determined after exposure to VOCs by SYST2 alone or in combination with *Rs*. Similarly, the relative expression levels of *RRS1* **(d)** *, Pr1a* **(e)** and *Pr1b* **(f)** were determined after exposure to synthetic VOCs. Error bars indicate standard errors of the means. Different lower case letters above the error bars represent significant differences according to Duncan’s multiple-range test (*P* ≤ 0.05) using SPSS software (SPSS, Chicago, IL)

To investigate whether the induced systemic resistance and growth promoting activity of VOCs is related to alteration in the relative transcriptional expression of genes, we examined the transcriptional levels of some key genes related to expansin (*NtEXPA1* and *NtEXPA2)*, ethylene (*ACO-1*), resistance (*RRS1*) and pathogenesis-related proteins (*Pr1a* and *Pr1b*). Samples were taken at 7, 14, and 21 days after exposure to VOCs produced by SYST2, total RNA was extracted, and the first-strand cDNA was synthesized from the polyadenylated mRNA. qRT-PCR assays performed with gene-specific primers demonstrated that SYST2-VOCs changed the relative expression of all the tested genes to varying levels (Figs. 8 and 9). The relative expression of the two expansin genes *NtEXPA1*and *NtEXPA2* was clearly increased after exposure to SYST2-VOCs, compared to control. However, increase in expression level was less when plants were exposed to SYST2-VOCs along with *Rs*-VOCs. Our results showed a clear down-regulation of the *ACO-1* gene after exposure to SYST2 VOCs alone or in combination with *Rs*-VOCs, at varying levels, compared to the control. *ACO-1* encodes ACC oxidase, a key enzyme that catalyzes the final step in ethylene biosynthesis. No significant effect was observed on the relative expression of these genes in water control and DH5α control. Similarly, albuterol and 1,3-propanediol up-regulated both the *NtEXPA1*and *NtEXPA2* while down-regulated the *ACO-1* (Figs. 8 and 9). Furthermore, we analyzed the genes of tobacco plant relating to resistance and defense by real-time PCR, to verify the resistance in tobacco against *Rs,* due to the exposure of VOCs. The transcriptional expression of R gene *RRS1,* involved specifically, in wilt resistance in tobacco against *Rs* was induced by the exposure to SYST2-VOCs and also by individual chemicals; albuterol and 1,3-propanediole compared to untreated control. However, over-expression was more by SYST2-VOCs as compared to individual chemicals and increased with the time, displaying its maximum at 9th days after inoculation. An up-regulation of defense-related proteins *Pr1a* and *Pr1b* genes was also observed after exposure to SYST2-VOCs. However, there was the difference in the relative expression levels of *Pr1a* and *Pr1b* among the VOCs produced by SYST2 and specific individual VOCs. Increased expression of *Pr1a* and *Pr1b* was noticed when treated with SYST2-VOCs compared to individual VOCs, which reached highest at 9th days (Figs. 8 and 9). Our result revealed that VOCs stimulated the resistance and defense-related genes which resulted in the induction of systemic resistance in tobacco against *Rs*.

## Discussion

Volatiles produced by microorganisms have been reported to have a valuable environment friendly role, in the form of beneficial interactions among PGPRs and plants, resulting in the induction of systemic resistance against biotic and abiotic elements [11, 17, 29], plant growth promotion [18, 23] and inhibition of plant fungal and bacterial pathogens [30]. Most of the previous studies on plant growth regulation and induced systemic resistance by bacterial VOCs described the interaction between VOCs produced by PGPR strain and plants only [12, 15, 21, 27]. In this study, we clearly demonstrated the interaction among PGPR strain *Bacillus subtilis* SYST2, plant pathogenic bacteria *Ralstonia solanacearrum* TBBS1 and tobacco plant. Both the beneficial and pathogenic bacterial strains coexist in soil and their efficiency as a PGPR or pathogen depends on their populations, nutrition and environmental conditions. In soil, many interactions coexist that affect each other’s biological activity. In this research, we choose to study the interactive effects of volatile compounds (VOCs) produced by both PGPR strain and pathogen *Rs*. A significant difference was observed in all growth parameters (fresh weight, dry weight, and leaf area) when tobacco seedlings were exposed to only SYST2-VOCs or individual compounds, albuterol and 1,3-propanediol in all experimental systems. No significant difference was observed following exposure to VOCs produced by pathogenic bacteria *Rs*. However, *Rs*-VOCs decrease the growth promoting potential of SYST2-VOCs, as a clear difference was observed when tobacco seedlings were exposed to only SYST2-VOCs or SYST2-VOCs in combination with *Rs*-VOCs. This decrease in growth-promoting potential of SYST2-VOCs suggests the growth inhibiting effect of *Rs*-VOCs against PGPR strain SYST2. To determine the interaction between *Rs*-VOCs and SYST2-VOCs, we inoculated SYST2 and *Rs* alone, and in combination, using I-plate system. Both SYST2-VOCs and *Rs-*VOCs inhibited the growth of each other but at different levels. However, SYST2-VOCs inhibited the growth of *Rs* more (56%) as compared to *Rs*-VOCs which inhibited the growth of SYST2 up to 22% (Fig. 5), suggesting the possibility that *Rs*-VOCs might be involved in decreasing the growth-promoting potential of PGPR strain SYST2 by inhibiting its growth. Most prior studies used I-plate system for the evaluation of the effect of VOCs on plant growth promotion or induced systemic resistance. MS-media is being used for plant growth in I-plates and due to its solid or semi-solid nature; VOCs only can reach the leaves while roots remained unexposed. Another point is that when we inoculate pathogen to evaluate induced systemic resistance, pathogen grows over the surface of MS-media and contaminates the I-plate. In this study, we modified the method, using “two plate system” in which soil mixed with vermiculite was used as a medium for plant growth. In this system, VOCs could reach the roots of seedlings through small pores present in the soil-vermiculite mixture. A significant increase in fresh and dry weights of tobacco was observed in this system following exposure to SYST2-VOCs; however, this increase was less (3.5-fold) as compared to I-plate system (7.7-fold). This difference might be due to unequal conditions for growth of seedlings in MS medium or soil medium, as MS medium is better for plant growth. Growth conditions in vitro and *in planta* are relatively different in terms of growing media and the degree of interaction of the tobacco with numerous environmental factors. PGPR live in soil naturally, and under natural conditions, the VOCs produced by PGPR usually interact with plant roots rather than leaves. Therefore, we moved the experiment from in vitro to pots in a growth chamber in order to determine the growth promoting activity by SYST2-VOCs in soil to expose the roots to VOCs. Our results revealed that SYST2 VOCs stimulated the growth under soil conditions also, which support the results of Park et al. [18].

Volatile organic compounds produced by microorganisms induced several physiological alterations relating to growth hormones and photosynthesis [24]. Our results showed a significant increase in photosynthesis rate, following the exposure to SYST2-VOCs. *Bacillus subtilis* GBO3-VOCs have been reported to enhance photosynthetic rate by increasing endogenous content of chlorophyll [31] and by changing the transcription of chloroplasts related genes, suggesting a correlation between the increase in photosynthetic activity and plant growth enhancement [32]. Up-regulation of transcriptional expression of the expansin genes *EXPB1, EXPB3, EXP4*, and *EXP5* in *Arabidopsis* [24], *EXP1, EXP2*, and *EXP6* in *Nicotiana* [5, 33], and *EXPA5* in *Lactuca sativa* [34] resulted in cell expansion. *Nt-EXPA1* and *Nt-EXPA2* encode tobacco expansin proteins that stimulate cell division and extension by loosening cell walls while *ACO1* gene (1-aminocyclopropane-1-carboxylic acid oxidase) is an important enzyme which regulates the ethylene production [35]. Our results showed that SYST2 VOCs up-regulated the transcriptional expression of the expansin-related genes *Nt-EXPA1* and *Nt-EXPA2* while a down-regulation was observed in ACO1. Our findings support formerly reported studies that bacterial volatiles has the potential to alter the transcriptional expression of genes relating to ethylene biosynthesis (*ACO2, ACS4, ACS12,* and *SAM-2*) and ethylene response (*CHIB, ERF1,* and *GST1*) [36]. Our results showed that SYST2-VOCs or synthetic VOCs, albuterol and 1,3-propanediole negatively influenced the wilt disease development by activating the induced systemic resistance in tobacco plants against bacterial wilt pathogen *R. solanacearum* TBBS1. However, induced systemic resistance appeared to be greater under in vitro conditions as compared to *in planta.* The difference observed could be due to the larger volume of the glass jars resulting in a lower concentration and availability of VOCs to the seedlings in the pots. In our previous experiment, synthetic VOCs, albuterol and 1,3-propanediole have been proved to induce plant growth in tomato. As we described above that SYST2-VOCs inhibited the growth of pathogen along with plant growth promoting activity. Although, *Rs*-VOCs also inhibited the growth of SYST2, but to a much lesser extent. In this context, SYST2-VOCs might be influenced in two positive ways: inhibiting pathogen growth and inducing systemic resistance, resulting in better plant growth. Our results suggested that SYST2-VOCs can elicit plant defense mechanisms. Molecular studies revealed that, resistance occurs as a result of an increase in the concentration of metabolites and defense-related enzymes, including PAL. The enzyme PAL has a significant role in the regulation of lignin accumulation and the creation of defensive structures, besides with the production of phenols which act as chemical defenses [37, 38]. PAL is also involved in plant salicylic-acid-mediated defense against plant pathogens while PPO catalyzes the oxygen-dependent oxidation of phenols. Both PPO and PAL participate in resistance mechanism against pathogenic microbes [39, 40]. Our results revealed an increase in both PPO and PAL in response to pathogen attack when plants were exposed to VOCs. These results are in conformity with previous results as a rise in the activities of PAL, PPO and PO enzymes was observed in *Bacillus*-treated tomato plants leading to the reduction of *Fusarium* wilt [41]. The results showed the up-regulation of transcriptional expression of R gene *RRS1* along with the overexpression of *Pr1a* and *Pr1b* when exposed to VOCs after inoculation with *Rs.* The resistant gene *RRS1* is specifically the bacterial wilt resistance gene in tobacco and the up-regulation of *RRS1* confirmed the activation of induced systemic resistance in tobacco [42]. The up-regulation of R gene (RRS1-R) in Col plants exhibited a clear increase in resistance level against wilt disease [43, 44]. Similarly, overexpression of R gene, *RE-bw* in eggplant, resulting in increased resistance, proved that the *RE-bw* was specifically a bacterial wilt resistant R gene. The up-regulation of transcriptional expression of *Pr1a* and *Pr1b* suggested that SA signaling-path way, might be involved as *PR1a* is activated during SA-dependent signaling pathway. Our results suggest that ISR can be achieved without any contact between PGPRs and plants, demonstrating that VOCs might be involved usually in the process of induced systemic resistance [45, 46].

## Conclusion

Our findings have shown that SYST2-VOCs significantly stimulated plant growth promotion and induced systemic resistance as well. Albuterol and 1,3-propanediol were found to be the key factors for growth promoting and ISR activity. SYST2-VOCs inhibited the growth of *Rs* and vice versa but inhibition of *Rs* by SYST2-VOCs, appeared to be much greater, resulting in the suppression of *Rs* inoculum level. Exposure to SYST2-VOCs or individual compounds altered the expression of genes involved in expansin, ethylene production, resistance and pathogenesis-related proteins.

## Methods

### Bacterial strain, plant material, and growth conditions

Tobacco (*Nicotiana tabacum*) seeds and both bacterial strains, *Bacillus subtilis* SYST2 (Accession no. GU568180.1), and *Ralstonia solanacearum* TBBS1 (Accession no. KY003096) used in this study, were obtained from our lab (Lab of biocontrol and bacterial molecular biology lab, Nanjing Agriculture University Nanjing). SYST2 was grown on Luria-Bertani (LB) medium at 37 °C overnight and stock cultures were maintained in LB broth supplemented with 30% glycerol at −20 °C. *Ralstonia solanacearum* TBBS1 was grown in tetrazolium chloride (TZC) agar medium (added 0.05% TTC) [47] for 48 h at 28 °C. “The stock” of *Rs* was preserved in sterilized distilled water at room temperature. Single colonies with a pink center were transferred to Casamino Acid Peptone Glucose (CPG) agar medium for experimental use [48]. Tobacco seeds were surface-sterilized by soaking in 70% ethanol for 1 min in an Eppendorf, followed by soaking in sodium hypochlorite (30%) for 15 min, rinsed four to five times in sterile, distilled water and dried on filter paper. The sterilized seeds were placed on Petri plates containing half-strength Murashige and Skoog salt (MS) medium [49], having 0.8% agar and 1.5% sucrose with 5.7 pH value.

### Growth-promoting activity of VOCs by SYST2 in vitro and *in planta*

For in vitro assays, two types of experimental systems were designed; “divided plate system” and “two Petri plate system”. For divided plate system, seven germinated seedlings were transplanted in one partition and bacteria were spot inoculated on the other partition. For “two plate system” we used soil as a medium for plant growth. A small plate (160 mm × 20 mm) having minimal salt media for bacterial growth was placed in a large plate (50 mm × 15 mm) having plant growth media (40% organic matter mixed soil +60% vermiculite). Seven equal size germinated seedlings were transplanted in the large plate containing soil mixture while bacteria were spot inoculated in the small plate. Plates were double sealed and incubated for further 10 days at 25 °C under a 12 h light/12 h dark photoperiod. The effect of VOCs produced by SYST2 on plant growth was determined by evaluating the differences in fresh green and dry weights.

For *in planta* assay, SYST2 was inoculated on a Petri plate placed at the bottom of a tissue culture jar (12 × 10 cm) as described by Park et al. (2015) [18]. Five germinated tobacco seedlings of equal size were transferred to the plastic pots (6 × 3 cm) containing soil (appropriate amounts of sand, clay, and organic matter), fixed on the glass jars and sealed with Parafilm to avoid the escape of VOCs produced by bacterial strains (SYST2 or DH5α). Five or six small holes (2 mm) were made in the bottom of the pots to allow the roots to be exposed to the VOCs. The pots fixed on the jars were then placed at 28/22 °C day/night under 16/8-h light/dark photoperiod. Plant growth enhancement was determined by evaluating the differences in fresh green weight, dry weight and leaf area between the controls and plants exposed to VOCs.

### Effect of volatiles produced by pathogenic bacteria on plant growth and efficiency of PGPR strain SYST2 in vitro and *in planta*

For in vitro assay, we used three partitioned plates, having MS media in one partition for plant growth and minimal salt media in other two partitions for inoculation of PGPR strain SYST2 and pathogenic strain *Rs*. Five germinated seedlings were transplanted in one partition, *Rs* was spot inoculated in 2nd partition while SYST2 was spot inoculated in the third partition. One set of plates was inoculated with only SYST2 and one set with *Rs* only while sterilized water was used in the third partition. In control plates, only germinated seedlings were placed. Plates were sealed and incubated as described earlier and data was taken after two weeks. To evaluate the effect of VOCs produced by pathogenic bacteria on plant growth and efficiency of SYST2 *in planta*, we placed two Petri plates each inoculated with SYST2 or *Rs* in one jar. In other two jars, SYST2 or *Rs* was spot inoculated in Petri plates, placed individually in each jar while DH5α or only water was used in controls. Five germinated tobacco seedlings of equal size were transplanted into the pots as described above and placed at 28/22 °C day/night under 16/8-h light/dark photoperiod. Fresh green weight, dry weight, and leaf area were measured after 28 days.

### GC-MS profile of VOCs produced by pathogenic bacteria *Rs*

A 20-μl suspension of overnight grown *Rs* cells was inoculated to 30 ml of MS agar medium in a 100-ml vial at 28 °C. A 2-cm divinyl benzene/carboxen /PDMS (DCP, 50/30 μm) solid phase microextraction (SPME) fiber (Supelco, Bellefonta, PA, USA) was used for collection of VOCs. After three days, the SPME fiber was inserted into the headspace of the vial containing bacteria and incubated at 50 °C for 30 min. The GC-MS analysis was performed using a Bruker 450-GC gas chromatograph complemented with a Bruker 320-MS mass spectrometer as described by [50]. Helium gas was used as the carrier at a flow rate of 1 ml min^−1^. The SPME fiber was desorbed at 220 °C for 5 min, and GC–MS was run for 25 min. The starting temperature of the column was 35 °C for 3 min, which was increased to 180 °C at a rate of 10 °C/min, further increased to 240 °C at 4 °C/min, and then held for 5 min. The mass spectrometer was operated in the electron ionization mode at 70 eV with a source temperature of 220 °C, with continuous scanning from 50 m/z to 500 m/z. The data in the NIST/EPA/NIH Mass Spectrum Library (Agilent Technologies, Santa Clara, CA, USA) was used for comparison and analysis to identify the compounds [10].

### Effect of VOCs produced by *Rs* on the growth of PGPR strain SYST2

To evaluate the effect of *Rs-*VOCs on the growth of SYST2, I-plate system was used which consisted of centrally partitioned plastic Petri dishes (85 × 15 mm) with no physical contact between the two microorganisms grown on either side. Ten μl of (18–24 h) *Rs* culture (1 × 10^7^ CFU ml^−1^) grown in CPG broth was dropped in one partition while 10 μl of liquid overnight-grown culture of SYST2 (1 × 10^8^ CFU ml^−1^) was dropped in the other partition containing modified minimal salt medium (MS) (1.5% agar, 1.5% sucrose, and 0.4% TSA (*w*/*v*)). The plates were double sealed and incubated at 28 °C for five days. Only pathogen and only SYST2 were inoculated on control plates. The diameter of *Rs* and SYST2 were measured in mm and viable cells were also counted by the 10-fold serial dilution technique. Each experiment was performed with five replicates, and the experiment was repeated three times.

### Effect of VOCs produced by SYST2 on activation of induced of systemic resistance in tobacco against bacterial wilt

To investigate the effect of VOCs on wilt disease development, the experiment was conducted both in “two plate system” and in plastic pots fitted on jars. Seven germinated tobacco seedlings were dipped in the suspension of *Rs* (1 × 10^7^ CFU/ml) cells and then transplanted into the large plate containing soil mixture while A 20-μl (10^8^ CFU/ ml) suspension of overnight grown PGPR strain SYST2 or synthetic chemical (albuterol and 1,3-propnaediole) was dropped into the small plate. The VOCs Albuterol and 1,3-propnaediole were identified from bacillus strain SYST2 using GC-MS analysis, in our previous experiment, and were proved as significant plant growth promoting VOCs [11]. For individual VOC evaluation, 50 μl of each chemical with two different concentrations, i.e., 1 mM albuterol, 0.1 mM albuterol 10 mM 1,3-propanediol and 1 mM 1,3-propanediol were used in one compartment, while in other compartment, tobacco seedlings were grown as described earlier. After one week of exposure to VOCs, wilt symptoms were observed and the data were recorded using the formula below. For *in planta* assay, plastic pots were fixed on glass jars (60 × 120 mm) and sealed with Parafilm to avoid the escape of VOCs from the jar, and a Petri plate (35 × 12 mm) was placed at the bottom of the jar. Five or six small holes (2 mm) were made in the bottom of the pots to allow the roots to be exposed to the VOCs. Five germinated tobacco seedlings were transplanted in each pot. After 1 week of plant growth, SYST2 or DH5α (20 μl, 10^−7^ cfu/ml) each was spot inoculated on Petri plates. DH5α and sterilized water were used as a control. To test the effect of synthetic VOCs in pot experiment, 100 μl of each compound with two different concentrations, i.e., 1 mM albuterol, 0.1 mM albuterol 10 mM 1,3-propanediol and 1 mM 1,3-propanediol were applied to small plates, that were placed at the bottom of jars. All pots were inoculated with a suspension of *Rs* (at an OD of 0.1 at 600 nm) by dipping the roots in the suspension, except the non-inoculated control, and then the seedlings were re-planted in the pots. The pots were placed in a growth chamber at 28/22 °C day/night temperature for a 16/8-h light/dark photoperiod at 85% relative humidity. The data for wilt was collected after 21 days of exposure to bacterial VOCs or synthetic VOCs, using the following formula: Disease index (%) *=* [*Σ* (*ni × vi*) ÷ (*V × N*)] × 100, where *ni* indicates the number of plants with the respective disease rating; *vi* = disease rating; *V* = the highest disease rating (5) and *N* = the number of plants observed. The disease rating was calculated using the scale: 1 = no symptoms, 2 = one leaf wilted, 3 = two to three leaves wilted, 4 = four or more leaves wilted and 5 = whole plant wilted. The experiment was performed using a completely randomized design with five replicates, and the whole experiment was repeated thrice.

### Polyphenol Oxidase (PPO) and Phenylalnine ammonia Lyases (PAL) activity

Five germinated tobacco seedlings were transplanted in each pot. After 1 week of plant growth, SYST2 (20 μl, 10^−7^ cfu/ml) was spot inoculated on Petri plates. DH5α and sterilized water were used as a control. After 10 days of VOCs exposure we inoculated a set of plants with *Rs* while control plants were inoculated with sterilized water only. Leaf samples were collected 0 (pre-inoculation), 24, 48, 72, 48, 72, 96 and 120 h after inoculation of *Rs*. Samples were stored directly in liquid nitrogen prior to analysis. For PPO extracts, 1.0 g of leaf tissue samples were taken, homogenized in 5 mL of phosphoric acid extracting buffer (0.05 M phosphate, pH 7.0), filtered and centrifuged at 12,000 rpm for 20 min. The whole process was performed at 4 °C using ice and the supernatants were collected for enzyme activity analyses. A similar extraction process was used for PAL extraction except for the buffer solution (0.05 M boric acid, 5 mM mercaptoethanol, 1 mM EDTA and 0.05 g of polyvinylpyrrolidone). The enzyme activity was evaluated as described by Li [51].

### Effect of SYST2-VOCs on transcription of genes involved in growth regulation and systemic resistance

Total RNA was extracted from leaf samples using TRIzol reagent (Invitrogen Biotechnology Co., Carlsbad, CA, U.S.A.) according to the manufacturer’s instructions. To determine the effect of SYST2-VOCs on transcription of genes involved in growth regulation, samples were taken at 7, 14 and 21 days exposure to VOCs while to determine the effect of VOCs on transcription of resistance related genes, samples were taken at 3, 6 and 9 days after inoculation of the pathogen. First-strand cDNA was synthesized using reverse transcriptase (TaKaRa Bio Inc., Tokyo, Japan) and random hexamer primers. Real-time PCR was performed using SYBR Green/Fluorescent qPCR master mix (Takara) on a Roche-480 system (Roche) using the *EF-1α* gene [52] as an internal reference. The transcriptional expression levels of resistance related genes *RRS1,Pr1a* and *Pr1b1* while growth regulation-related genes (NtEXPA1, NtEXPA2, and ACO1) were detected. The qRT-PCR program consisted of denaturation at 95 °C for 1 min, followed by 40 amplification cycles at 95 °C for 5 s, 57 °C for 30 s, and 72 °C for 30 s. The specific primers used in this study are listed in Additional file 1: Table S1. Each sample was replicated thrice for qPCR, and 2 − ΔΔCt method was used to analyze gene expression level [53].

### Statistical analysis

To evaluate the significance of the treatments, the data from each experiment was analyzed using analysis of variance (ANOVA) and Duncan’s multiple-range test was employed to assess differences among treatments at *P* = 0.05 using SPSS ver. 17.0 statistical software (SPSS, Chicago, IL). Graphs and figures were plotted using sigma plot version 10.0.

## Additional file

Additional file 1: Primers used in this study and VOCs produced by *Ralstonia solanacearum*. (DOCX 34 kb)

## Abbreviations

CPG: Casamino Acid Peptone Glucose
DOVE-MO: Data of volatiles emitted by microorganisms
ISR: Induced the systemic resistance
LB: Luria-Bertani
MS: Murashige and Skoog
PAL: Phenylalnine Ammonia Lyases
PGPR: Plant growth-promoting rhizobacteria
PPO: Polyphenol Oxidase
Rs: *Ralstonia solanacearum* TBBS1
SPME: Solid phase microextraction
TZC: Tetrazolium chloride
VOCs: Volatile organic compounds
