# Supplementary material for: Effect of volatile compounds produced by Ralstonia solanacearum on plant growth promoting and systemic resistance inducing potential of Bacillus volatiles
Source: BMC Plant Biol. 2017 Aug 2;17:133. doi: 10.1186/s12870-017-1083-6 (PMC5541429; doi:10.1186/s12870-017-1083-6)
Supplement: Additional file 1: — Primers used in this study and VOCs produced by Ralstonia solanacearum. (DOCX 34 kb) [file 12870_2017_1083_MOESM1_ESM.docx]

**Supplementary Information**

Hafiz Abdul Samad Tahir^1,2^, Qin Gu^1^, Huijun Wu^1^, Waseem Raza^1^, Asma Safdar^1^, Ziyang Huang^1^ Faheem Uddin Rajer and Xuewen Gao^1*^

^1^Department of Plant Pathology, College of Plant Protection, Nanjing Agricultural University, Key Laboratory of Integrated Management of Crop Diseases and Pests, Ministry of Education, Nanjing 210095, PR China

^2^Plant Pathology section, Tobacco Research station, Pakistan Tobacco Board, Ministry of Commerce, Govt. of Pakistan, Hayatabad, Peshawar, Pakistan.

^*^ Corresponding author: Xuewen Gao

Address: Nanjing Agricultural University, Weigang No.1, Nanjing 210095, PR China

Email: gaoxw@njau.edu.cn

Telephone/Fax: 86-25-8439526

**Table S1: Primers used in the study**

| **No** | **Oligo Name** | **Sequence 5^/^ to 3^/^** |
| --- | --- | --- |
| 1 | *EF-1α (F)* | AGACCACCAAGTACTACTGCAC |
| 2 | *EF-1α (R)* | CCACCAATCTTGTACACATCC |
| 3 | *Nt-EXPA1-F* | GGTGGTATTGTTCCTGTC |
| 4 | *Nt-EXPA1-R* | CCAGCCCCTCCTACATTGCTTATCA |
| 5 | *Nt-EXPA2-F* | CCTAAATGGTGTAGAAAAG |
| 6 | *Nt-EXPA2-R* | AATGCCACCTCTGTAAAT |
| 7 | *ACO1-F* | CTTCCAAGATGACAAAGTAAGCGGC |
| 8 | *ACO1-R* | ACGACGATGGAGTGGC |
| 9 | *RRS1(F)* | ATGAGAAAGAGGCTCGTCAA |
| 10 | *RRS1(R)* | ACCACAACCCTCAAGCAGTT |
| 11 | *Pr1a (F)* | ATGCGCAAAATTATGCTTCC |
| 12 | *Pr1a (R)* | CCTAGCACATCCAACACGAA |
| 13 | *Pr1b (F)* | GCAGACTGCAACCTCGTACA |
| 14 | *Pr1b (R)* | CCTAGCACATCCAACACGAA |

**Table S2: VOC profile of *Ralstonia solanacearum***

| **RT (min:s)** | **Total area (%)** | **Possible compounds** | **CAS No.** | **Abbreviation used** | **Degree of inhibition** |
| --- | --- | --- | --- | --- | --- |
| 8.71 | 5.06 | Cyclotetrasiloxane, octamethyl- | 5567-67-2 | CTS | _ |
| 8.90 | 1.39 | 1,1,3,3,5,5,7,7-Octamethyl-7-(2-methylpropoxyl) tetrasiloxane-1-ol | 193407 | OTS |  |
| 9.50 | 1.23 | Sulfurous acid, cyclohexamethyl hexyl ester | 6349 | SCE | - |
| 10.3 | 1.54 | Dichloroacetic acid, 2-ethylhexyl ester | 86144-72-9 | DCA | +++ |
| 10.38 | 8.34 | Dodecane, 1-fluoro | 334-68-9 | DFC | + |
| 11.06 | 3.42 | Cyclopentasiloxane, decamethyl | 541-02-6 | CPS | - |
| 13.40 | 1.69 | 2-Tetradecanone | 2345-27-9 | 2-TD | ++ |
| 13.51 | 4.16 | Cyclohexasiloxane, dodecamethyl | 540-97-6 | CHS | _ |
| 14.2 | 1.76 | Phenole, 2-(1,1-dimethylethyl)-4-methyl | 2409-55-4 | PH | - |
| 14.87 | 1.09 | Tridecane | 629-50-5 | TDC | _ |
| 16.1 | 1.21 | Hexadeacne |  | HDC | + |
| 18.65 | 1.112 | Butanamide | 97443-86-2 | BMD | ++ |

Similar compounds present both in inoculated and non-inoculated MS medium were excluded. Several minor air-peaks (≤1% of the total area ) were also not included. RT, retention time. +++ = more than 30 % inhibition, ++ = 20 to 30 % inhibition, + = 5- 10 % inhibition
